# Exploring the associations between resilience and psychological well-being among South Africans during COVID-19

**DOI:** 10.3389/fpsyg.2024.1323466

**Published:** 2024-02-13

**Authors:** Tasleem Sayed, Hanelie Malan, Erika Fourie

**Affiliations:** ^1^Community Psychosocial Research (COMPRES), Faculty of Health Sciences, North-West University (NWU), Potchefstroom, South Africa; ^2^Research Design, Statistical Analysis and Interpretation: Pure and Applied Analytics, School of Mathematical and Statistical Sciences, North-West University (NWU), Potchefstroom, South Africa

**Keywords:** COVID-19, pandemic, positive mental health, psychological well-being, resilience

## Abstract

Resilience pertains to an individual’s ability to withstand, adapt, and recuperate from adversity and stress. As the world grapples with unprecedented challenges such as the COVID-19 pandemic, understanding the relationship between resilience and psychological well-being becomes essential. Preliminary observations suggest that those with a higher resilience tend to have better psychological well-being, indicating a possible symbiotic relationship between the two. This study was structured using a cross-sectional survey design. A convenience sampling technique was employed, including 631 respondents in South Africa. Data collection took place between June 11 and July 9, 2022, facilitated through a Google Forms questionnaire. This questionnaire encompassed various instruments, namely a biographical questionnaire, the CD-RISC 10, the WHO Well-being Index, the FACIT-Sp-12, and the PMHS. The findings from the collected data highlighted a strong correlation between resilience and overall well-being during the COVID-19 pandemic. This elevation in resilience can be instrumental in augmenting psychological well-being. As such, interventions or programs aimed at enhancing individual and community well-being might benefit from incorporating elements that bolster resilience, especially during periods of global adversity.

## Introduction

During the COVID-19 pandemic, countless South Africans found themselves isolated at home, trying to navigate the pandemic with the strengths and resources they had. The aftermath of the pandemic has seen an increase in anxiety and depression among individuals of all ages. The ongoing pandemic has created an unprecedented disaster, profoundly altering daily life for many people, such as increased uncertainty, fear, illness, and death; a rise in a variety of stressors; and reduced access to protective factors.

Pandemics are typically rare and unpredictable, and little preparation is made to navigate physical and mental health repercussions. During the COVID-19 pandemic, people had to adapt their daily routines to accommodate new developments and threats to their health, social networks, employment, means of subsistence, and education (Rolland, 2020). Families struggled to make sense of the pandemic experience, coping with huge uncertainty, and adjusting to changes that included the loss of expectations, hopes, and previous ways of life (Walsh, 2020). Although the majority of individuals have suffered devastating consequences from the pandemic, studies have shown that individuals can recover and adapt to pandemic-related stressors (Yıldırım et al., 2022). This adaptability can be attributed, in part, to positive mental health. According to Lukat et al. (2016) and Ngoc Nguyen et al. (2022), positive mental health encompasses several intersecting threads that build an individual, especially during times of distress and difficulty; it is a key asset and resource in well-being. These threads can include a variety of internal and external protective factors that are concerned with the quality of a person, their environment or their interactions. These elements, which are predictive of improved outcomes (Rojas Flóres, 2015), may enhance resilience (Pillay, 2020) in an attempt to promote psychological well-being.

The study of positive outcomes and flourishing after adversity in the field of Positive Psychology has attracted much attention in recent years. Peterson and Seligman (2003) explain that traditional psychology has focused on identifying and treating human ills; however, positive psychology pays attention to people’s strengths while also acknowledging their weaknesses and fosters well-being while not ignoring the healing of distress. Seligman (2011) explains that the field of Positive Psychology encompasses more than simply the study of dysfunction and mental illness; it also investigates the positive qualities that contribute to health and well-being (Snyder and Lopez, 2002). The power of positive psychology is its ability to apply the most successful aspects of individuals to their unique situations. With this in mind, recent advances in positive psychology have advanced studies on building resilience and promoting positive psychological functioning (Didkowsky and Ungar, 2016; Sandberg and Grant, 2017; Dubus, 2018; Gonzalez-Mendez et al., 2021; Boitshwarelo et al., 2022).

Researchers who examined resilience proposed a wide variety of philosophies and ideologies, each with their own body of evidence from which to construct a definition of resilience. While earlier studies on resilience described resilience as an inherent trait, Luthar (2006) and Rutter (1993) found that resilience was guided by factors other than merely a person’s character, including cultural and environmental factors. From this emerged the notion that resilience is a process (Ungar, 2011, 2018).

People either have resilience as a *trait* or not. However, the concept of resilience as a *process* implies that it may be fostered and enhanced. Masten (2007), Luthar et al. (2000) and Ungar (2011, 2018) added to the definition of resilience by describing it as the interaction of the individual with their environment that results in positive adaptation. Luthar (2003) stresses that resilience cannot be quantified or measured. Although there are several scientific and standardized instruments to measure resilience such as the CD-RISC (Davidson, 2020), Resilience Scale-14 (Wagnild and Young, 1993), Resilience Attitudes and Skills Profile (Hurtes and Allen, 2001), Resilience Scale for Adults (Friborg et al., 2003), Psychological Resilience Scale (Windle et al., 2008) and the Brief Resilience Scale (BRS) (Smith et al., 2008), these measurements measure the protective factors within the individual’s life to determine their level of functioning within their environment. How one develops resilience and the process for reaching a resilient outcome have been studied by Cavazos Vela et al. (2014), Rajan et al. (2017), Raniga and Mthembu (2017), and Finlay et al. (2021) in many contexts and across many various populations, and even though the definition of resilience still seems elusive, studies (Beames et al., 2021; Di Giuseppe et al., 2021) have determined factors associated with this significant construct. When confronted with a stressful or traumatic event, resilience allows people to cope with and adapt to challenging life situations. Resilience has the capacity for the individual to recover while interacting with protective factors within their environment; therefore, resilience is seen as an ordinary but powerful form of strength.

The concept of well-being is complex (Dodge et al., 2012; Huta and Waterman, 2014), and no single theory adequately explains it since the status of psychological well-being is shaped by many factors. Thus, researchers (Rahmani et al., 2018; Watts et al., 2022) and mental health professionals continue to study well-being in diverse populations to refine and grasp the concept of well-being. Early research on well-being by Ryff (1989) elucidates that psychological well-being is a multidimensional construct and can be viewed as positive psychological functioning. Recently, Ryan and Deci (2001) added that psychological well-being is a process of living and functioning well and reaching one’s full potential. Theories of Ryff (1989) and Ryan and Deci (2001) suggest that psychological well-being encompasses a broader range of factors that contribute to an individual’s overall mental health and life satisfaction, and this should not be confused with happiness. Happiness typically refers to a subjective emotional state characterized by positive feelings (Ruggeri et al., 2020). The architecture of psychological well-being does not only relate to happiness and feeling well but also to experiencing positive emotion, engagement, relationships with others, life meaning and achievement (Seligman, 2011).

There are two dimensions associated with psychological well-being, namely hedonic and eudaimonic well-being. Hedonic well-being is a subjective experience mostly related to pleasure and positive experiences; it is centered on experiencing positive emotions and minimizing negative emotions (Fredrickson, 1998; Ryan and Deci, 2001; Lyubomirsky et al., 2005). Hedonic well-being can be experienced through the satisfaction of immediate desires, such as feeling or experiencing pleasure in the current moment.

Eudaimonic well-being, on the other hand, refers to finding meaning and purpose in one’s life. This relates to living well (Baluku et al., 2022) and includes experiences and activities that promote self-actualization, personal meaning, reaching one’s purpose and personal goals and a sense of psychological well-being (Ryff, 1989; Ryan and Deci, 2001; Steger et al., 2008; Huta and Ryan, 2010). These activities or experiences then result in a feeling of meaning, fulfillment and flourishing, which relates to Aristotle’s ideas of striving for purpose and meaning (Ryff, 1989). Research suggests that resilience is closely related to hedonic and eudaimonic well-being (Ruggeri et al., 2020). Studies show that more resilient individuals tend to experience higher levels of psychological well-being (Smith and Hollinger-Smith, 2015) as well as optimal functioning (Di Fabio and Palazzeschi, 2015).

Since well-being is a complex phenomenon that includes optimal functioning and wellness (Ryan and Deci, 2001), efforts to promote well-being have increased. A wealth of research on the science of well-being identifies elements that contribute to it; elements such as resilience, for example (Di Fabio and Palazzeschi, 2015; Smith and Hollinger-Smith, 2015). The experience of positive emotions is also linked to a sense of well-being and life satisfaction (Quoidbach et al., 2010). Positive emotions such as hope (Pleeging et al., 2021) cultivate resilience and well-being in individuals alongside mindfulness (Robinson and Eid, 2017).

Resilience can help individuals adapt to stress and cope with difficult situations more effectively, which in turn can reduce anxiety levels. This suggests that the presence of resilience is an important factor for overall well-being and health. During the COVID-19 pandemic, individuals globally suffered from acute stress, anxiety and loneliness (Aluh and Onu, 2020; Polizzi et al., 2020; Sümen and Adibelli, 2021). Research during the pandemic has shown that individuals who possess higher levels of self-efficacy and optimism are more likely to exhibit greater resilience in the face of adversity (Robles-Bello et al., 2020). Miragall et al. (2021) found that resilience was positively correlated with meaning and gratitude. Having higher levels of self-efficacy and optimism can help individuals maintain a sense of control and hope in the face of uncertainty, which can contribute to their overall well-being.

The COVID-19 pandemic has been a significant challenge for many people around the world, and resilience has been an important factor in how individuals have coped with the various stressors and uncertainties associated with the pandemic. Some individuals have shown remarkable resilience during the pandemic, demonstrating the ability to adapt to changes, cope with stress, and maintain a positive outlook despite the challenges they face (Baños et al., 2021; Park et al., 2021). This resilience can be attributed to a variety of factors, such as character strengths (Casali et al., 2021), personal strength, spirituality (Li et al., 2022), and family support (Rich et al., 2022), among others. Thus, it can be assumed that resilience has been an important factor in how individuals have coped with the pandemic, and those who have shown high levels of resilience were able to adapt better to the challenges they face and maintain a positive outlook despite the difficulties.

Within the South African population amidst the COVID-19 epidemic, a heightened degree of resilience will have a favorable correlation with increased psychological well-being. More precisely, individuals with higher scores on resilience, indicating their capacity to adjust to stress, bounce back from negative events, and maintain a positive perspective, will also exhibit elevated levels of psychological well-being. This is evident through factors such as decreased symptoms of anxiety and depression, increased life satisfaction and enhanced overall mental health.

The researchers aimed to examine the relationship between resilience and psychological well-being of South Africans during the COVID-19 pandemic. The methods used to achieve this aim are discussed below.

## Methods

### Study design

This study was quantitative and adopted a cross-sectional survey research design. This type of design was suited for the nature of the study as the researcher wanted to collect data at a specific time during the COVID-19 pandemic from the South African population. With this, the purpose is to gather information about a specific variable or set of variables from a diverse group of respondents to gain insights into their characteristics, opinions, behaviors and experiences.

### Study context

South Africa has faced one of the world’s highest infection rates, with over 4 million confirmed cases and more than 100,000 deaths as of October 2023 (Worldometer, 2023). South Africa responded to the COVID-19 pandemic with prompt actions, including the implementation of an early nationwide lockdown and a comprehensive public health response, in alignment with the guidance of World Health Organization (WHO) (2021). The declaration of the State of Disaster by the President, per the Disaster Management Act DMA (2020), necessitated the implementation of national lockdown measures to curb the spread of the virus (World Health Organization (WHO), 2021; October et al., 2022).

### Respondents

**Figure 1:**
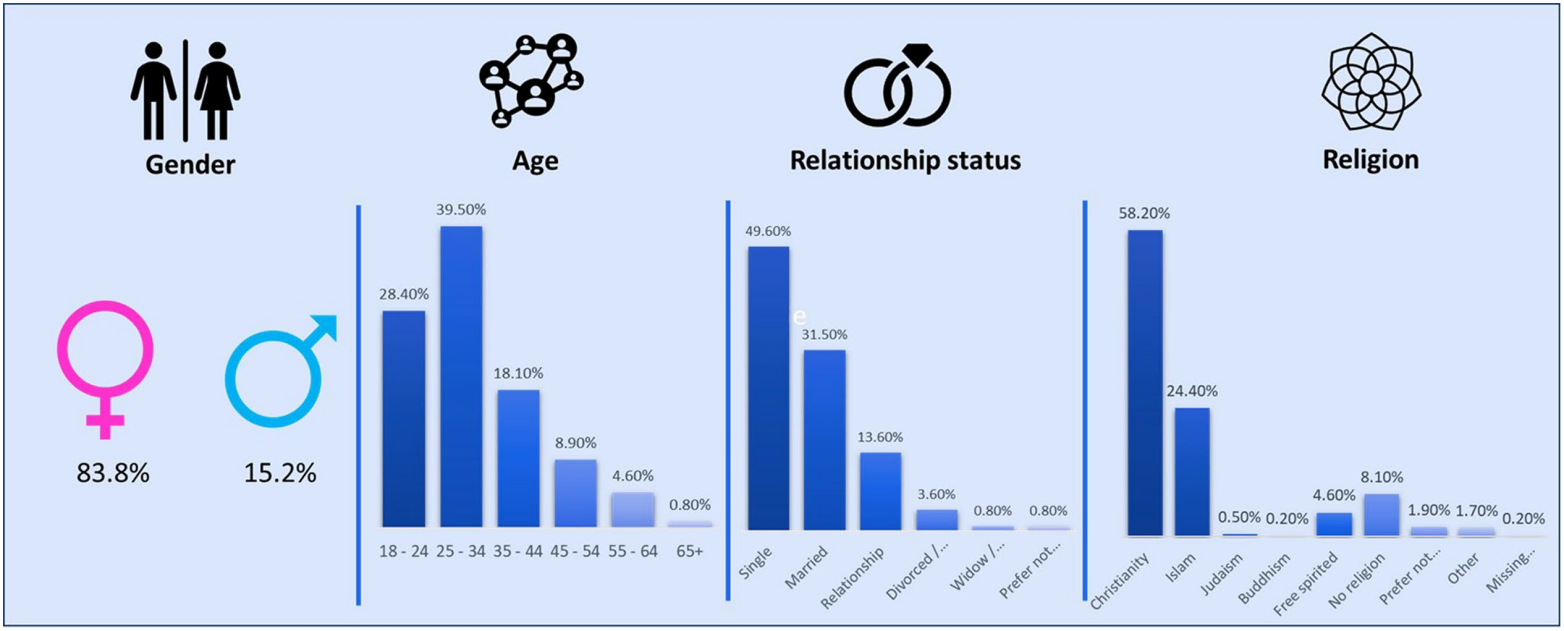
Sample demographics.

According to Figure 1, the research sample comprised 631 respondents from South Africa across all nine provinces in the country. Females represented most of the sample with a total of 83.8% (*n* = 529); males represented 15.2% (*n* = 96). A high number of respondents fit into the age category of 25–34 years of age. Almost half of the sample was single (49.6%). Christianity represented most of the sample with 58.2% (*n* = 367).

### Instruments

The instruments used to collect data were a biographical questionnaire, the Connor-Davidson Resilience Scale (CD-RISC 10), the World Health Organisation (WHO-5) Well-being Index, the Positive Mental Health Scale (PMHS), and the Functional Assessment of Chronic Illness Therapy-Spiritual Well-being (FACIT-Sp-12). These tools aimed to explore the associations between resilience and psychological well-being among South Africans during COVID-19.

#### Biographical questionnaire

A biographical questionnaire was prepared to capture information on the age, gender, location and religious affiliation of respondents. This was done to provide a comprehensive understanding of the study sample and their responses to the COVID-19 pandemic.

#### Connor-Davidson resilience scale

Resilience was measured with the CD-RISC 10. Responses are reported on a five-point Likert scale that ranges from 0 (not true at all) to 4 (true nearly all the time) and consist of a series of items that respondents assess according to their own experiences and emotions (Campbell-Sills and Stein, 2007). For this study, respondents were asked to answer the questions specifically about the COVID-19 pandemic. The questionnaire assessed both the individual’s state of resilience during the COVID-19 pandemic, their overall ability to cope with adversity as well as their capacity to cope with difficult life experiences.

The CD-RISC 10 has been widely employed in both research and clinical environments to assess resilience among diverse populations and within various contexts. Notably, studies investigating resilience during the COVID-19 pandemic have reported Cronbach’s alpha coefficients ranging from 0.88 to 0.94, indicating strong internal consistency (Kavčič et al., 2020; Mosheva et al., 2020; Cobo-Cuenca et al., 2022). This scale offers valuable insights into individuals’ capacity to effectively navigate and rebound from challenging life circumstances.

#### The World Health Organisation well-being index

The WHO-5 is a self-report questionnaire designed to assess subjective well-being that was developed as a unidimensional scale (Cosma et al., 2022). Respondents were asked to rate the applicability of each of the five statements to their experience during the COVID-19 pandemic (Staehr, 1998).

The WHO Well-being Index has been widely utilized in various countries (Carranza Esteban et al., 2022; Cosma et al., 2022; Kassab Alshayea, 2023) and contexts (Chan et al., 2022; Lara-Cabrera et al., 2022) to assess well-being and monitor changes over time. For the WHO-5, Cronbach’s alpha is between 0.8 and 0.9 in multiple studies (Lara-Cabrera et al., 2022; Low et al., 2023), suggesting high internal consistency. It provides a quick and reliable measure to evaluate an individual’s overall emotional well-being.

#### Positive mental health scale

The PMHS is a self-report unidimensional scale that is used to assess a person’s level of positive mental health (Lukat et al., 2016; Toledano-Toledano et al., 2023). For this study, an additional distinction was introduced in the questionnaire. Originally, Item 6 proposed, “I am in good physical and emotional condition”; however, this was divided into two separate statements: “I was in good physical condition” and “I was in a good emotional condition.” This adaption, while recognizing the reciprocal relationship between physical and emotional conditions, was designed to encourage respondents to give more thoughtful consideration to their specific emotional state (Lukat et al., 2016).

The internal consistency of the PMHS has been reported to be robust with Cronbach’s alpha values of 0.86 (Almubaddel, 2022), 0.87 (Velten et al., 2021), and between 0.92 (at Time 1) and 0.93 (at Time 2), according to Brailovskaia and Margraf (2020).

#### Functional assessment of chronic illness therapy-spiritual well-being

Spiritual well-being was measured with the FACIT-Sp-12 (Brady et al., 1999; Peterman et al., 2002), which is a 12-item measure with questions that assess three domains of spiritual well-being (Bredle et al., 2011). These three domains are Faith (Items 9, 10, 11, 12), Meaning (Items 2, 3, 5, 8), and Peace (Items 1, 4, 6, 7). EFA of the items revealed two or three factors (Monod et al., 2015; Damen et al., 2021). Most studies have used FACIT-Sp-12 in cancer and HIV patients. It has never been used in a COVID-19 setting before, but it can be adapted for use in the general population. The Cronbach’s alpha for the total scale of the FACIT-Sp-12, which measures the scale’s internal consistency, has been reported to be between 0.79 and 0.87 according to studies by Aktürk et al. (2017) and Rabitti et al. (2020).

### Procedure

The recruitment and compensation process for the study’s respondents were meticulously executed through well-planned steps. These measures not only ensured a robust response rate to the questionnaire but also facilitated smooth compensation for the respondents.

Initially, a data collection tool was created using Google Forms, encompassing a comprehensive online questionnaire with a biographical section and standardized scales. To maximize reach, this questionnaire was strategically disseminated across various social media platforms, including Facebook, Instagram, and X (previously known as Twitter). To further expand its visibility, an independent person was tasked with circulating the study’s advertisement across a range of South African Facebook groups, thereby reaching a diverse audience.

In addition, the National Research Foundation (NRF) played a pivotal role in promoting the study by broadcasting the advertisement through their X account, contributing significantly to the study’s outreach. Parallel to these efforts, a dedicated Instagram account named ‘covid-19 resilience’ was created. This account employed paid promotions as an effective strategy to further disseminate the advertisement and recruit respondents.

The survey link was prominently featured in the online advertisements and was active from June 11 to July 9, 2022. During this period, the questionnaire gathered a substantial number of responses, totalling 639. Following this successful response period, the researcher prudently closed the questionnaire to cease further responses. This closure marked the end of the data collection phase, setting the stage for the subsequent analysis and interpretation of the gathered data.

The advertisement and the questionnaire’s introductory page provided respondents with detailed information about the study’s purpose. The digital format facilitated informed consent via a clickable button on the electronic questionnaire. Clicking this button, after understanding the study details, signified the respondent’s willingness to participate and represented their formal consent to be included in the research. Once they consented, the respondents went on to answer the questionnaire, which took them 15–20 min to complete. Data were collected from June 11 to July 9, 2022. During the specified period, a total of 639 responses were collected. However, only 631 responses were included in this analysis. The excluded responses were due to incomplete questionnaires, one instance of non-provision of informed consent, which led to the discontinuation of the questionnaire, and an instance where a respondent filled out the questionnaire multiple times. Respondents of the survey were graciously compensated with a R50 (approximately $2.50) Clicks voucher, serving as a gesture of gratitude for their valuable time and effort in completing the questionnaire. To facilitate this token of appreciation, respondents who chose to receive compensation were requested to provide their cell phone numbers. Within a span of 30 days following their participation, a voucher from Clicks, a well-known local retail drugstore, was generated. This voucher was then promptly dispatched to the provided cell phone number of each respondent. This process not only ensured a seamless distribution of the vouchers but also reinforced the appreciation toward the respondents for their crucial contribution to the research.

### Ethical considerations

The HREC of the Faculty of Health Sciences at the NWU approved the study, with the ethics number NWU-00242-21-A1. To prioritize respondent privacy, the online questionnaire was structured to limit access to personal data. The only personal information obtained from the respondents was cell phone numbers, for the purpose to distribute tokens of appreciation.

### Data analysis

An EFA was performed on each of the different instruments used in this study. EFA is a statistical technique used to identify patterns and relationships among a set of variables (Finch, 2020). The EFA aims to identify the minimum number of factors that can account for the maximum amount of variation in the data. The result of an EFA is a set of factor loadings tabled within a pattern matrix, which indicates the strength of the relationship between each observed variable and each factor (Fabrigar and Wegener, 2011). These loadings can be used to interpret the underlying factors and identify which observed variables are most strongly related to each factor, such as under which factor the observed variable or item loads.

The principal component analysis method with the direct oblimin rotation method was used to perform factor analyses on the data. The KMO measure and Bartlett’s Test of Sphericity were conducted to explore the suitability of the data. According to Field (2009), the KMO is utilized to test for sample adequacy and results greater than 0.7 suggest that sample size is acceptable. Bartlett’s Test of Sphericity is a statistical test used to verify the appropriateness of performing a factor analysis on a particular dataset. Bartlett’s Test of Sphericity evaluates the null hypothesis, which posits that there is no significant correlation among the variables (Meyers et al., 2013). If Bartlett’s test is statistically significant (value of *p* < 0.05), it suggests that there are some relationships between the variables in the dataset that are suitable to be explored further with factor analysis. If the test is not statistically significant, it implies that the variables are unrelated and therefore factor analysis may not be appropriate or informative for the given dataset (Shrestha, 2021).

Communality, in the context of factor analysis, refers to the percentage of variance in a given item that can be accounted for by the extracted factors (Field, 2013; Tavakol and Wetzel, 2020). It represents how much of an item’s variation can be explained by the identified factors. If an item has a high communality, it means that it shares a large amount of variance with the other items on the scale and thus contributes to a greater extent to the overall construct being measured (Hair et al., 2019). Low values (less than 0.3), indicate that the item does not fit well with the other items in the component (Pallant, 2013). The closer communality values are to one, the less unique variance a variable has, which indicates that the factor structure is supported (Field, 2013; Tavakol and Wetzel, 2020). Hair et al. (2019) explain that 0.50 is recommended in a sample of over 300 individuals.

PCA is centered on elucidating the total variance encompassed by a set of variables. Within this framework, each variable contributes exactly 1,000 units, making the total variance equivalent to the number of variables involved in the analysis (Meyers et al., 2013). A goal of factor analysis is to explain as much of this total variance as possible with a smaller number of factors (Field, 2005). The more variables that load onto a particular component (in other words have a high correlation with the component), the more important the factor is in summarizing the data.

### Factor analysis and reliability

#### Connor-Davidson resilience scale

KMO and Bartlett’s Sphericity tests showed that the data were suitable for factor analysis (KMO: 0.946, Barlett’s Test of Sphericity, *p* < 0.0001). The communalities for the CD-RISC 10 ranged from 0.496 to 0.720, thus, the extracted factor explained between 49.6 and 72.0% of the variance in the observed variables (items) included in the scale. Higher communalities generally indicate better measurement properties, as they suggest that the items are more strongly related to the underlying construct being measured. The extracted factors explained 66.449% of the total variance (Campbell-Sills and Stein, 2007).

While Connor and Davidson’s (2003) early publications on the CD-RISC’s structure allowed for further analysis, subsequent research has cautioned against utilizing factor-level scoring on the CD-RISC’s subscales (Davidson, 2020). This may be because the factor structure of the CD-RISC may not be consistent across different settings, making it difficult to interpret scores on individual subscales. Therefore, for this study, only one factor was extracted. The Cronbach’s alpha value of 0.953 indicated a high level of internal consistency and reliability.

The application of the CD-RISC 10 in this study has demonstrated a moderate level of resilience among respondents, with a mean score of 25.39 (SD = 9.40). This score, above the midpoint of 20, suggested that, despite the challenges of the COVID-19 pandemic such as health concerns, social isolation, financial instability, and alterations in daily life (Petry et al., 2020; Goldberg et al., 2021; Yeung et al., 2022; Maragha et al., 2023), respondents showed a noteworthy level of resilience on average. When compared to other studies conducted during the pandemic using the CD-RISC 10, this manuscript’s findings were similar, with Aruta (2022) reporting a mean score of 28.8, and Kavčič et al. (2021) noting a score of 27.3. Therefore, the experience of moderate resilience was consistent across different samples during this challenging time.

#### World Health Organisation well-being index

A KMO value of 0.884, along with a statistically significant value of *p* of 0.001 for Bartlett’s Test of Sphericity, indicated that the dataset was suitable for conducting factor analysis. In this case, the range of communalities was 0.740–0.801 (Staehr, 1998). This reflects that the common factor underlying the set of variables used in the well-being index accounts for substantial amounts of the total variance of the individual variables. The total variance explained after extraction was 76.863%.

In comparison, a separate study conducted in New Zealand during the same pandemic period reported an overall mean WHO-5 score of 14.7 (SD = 5.71). This indicated that, on average, individuals in New Zealand had slightly higher subjective well-being compared to the sample in the current study; however, it still fell within the range of average well-being. These findings underscore the importance of considering regional and contextual factors when assessing well-being during challenging times.

#### The positive mental health scale

In the PMHS, originally consisting of nine items, a modification was made to Item 6 to enhance clarity (Lukat et al., 2016). More specifically, Item 6 “I was in a good physical and emotional condition” was split into two separate questions: “I was in a good physical condition,” “I was in a good emotional condition.” The first question assessed the respondent’s perception of being in a good physical condition, and the second question evaluated their perception of being in a good emotional condition.

For the PMHS, the KMO amounted to 0.955 and Bartlett’s Test of Sphericity was highly significant (*p* < 0.0001). This indicated that the variables in the data set were correlated enough to warrant the use of factor analysis.

In this case, the communalities for the PMHS ranged from 0.568 to 0.765, which indicated that between 56.8 and 76.5% of the items’ variances were explained when combining them all into one factor. The extracted factor explained 69.899% of the total variance. For this study, the Cronbach’s alpha was = 0.951. The mean factor score of 17.01 (SD = 9.91) out of a maximum of 40 indicated that the average level of positive mental health within the sample group was moderately low. This suggested that there was room for improvement in terms of positive mental health among the respondents. Given the scale’s focus on positive aspects of mental health such as PA, life satisfaction, and psychological functioning, the score reflected that the respondents in this sample may have been experiencing challenges in these areas. When comparing the results of this study to those of a Spanish sample reported by Boufellous et al. (2023), a noteworthy discrepancy emerged. The Spanish sample exhibited a notably higher mean score of 19.16 (SD = 6.42), even though they used a different maximum score of 27. This contrast suggested that, in comparison, the positive mental health of the respondents in this study may have been lower. These findings highlighted potential variations in mental health and well-being between the two groups, raising important questions about the factors that may contribute to these differences.

#### Functional assessment of chronic illness therapy-spiritual well-being

The FACIT-Sp-12 showed a KMO value of 0.87 and Bartlett’s Test of Sphericity was statistically significant (*p* = 0.001). Thus, the sample size was sufficient to carry out the factor analysis of the data (Bredle et al., 2011).

The items of the spiritual well-being questionnaire had communalities ranging from 0.54 to 0.90. However, two specific items—Question 4 and Question 8—demonstrated particularly low communalities of 0.093 and 0.176, respectively. This indicated that these two questions share a relatively smaller proportion of variance with the other items in the questionnaire. This could be because the two questions are negatively phrased. As per Table 1’s pattern matrix, two factors were extracted for the items in the questionnaire and the total variance explained by these factors was 62.93%. According to the literature, Question 4, “I have trouble finding peace of mind” loads under Factor 1 (Peace and meaning), but after careful consideration based on the expertise of the researchers, it was decided to group it under Factor 2 (Faith) as indicated by the results of the EFA. Specifically, studies on spirituality, faith and peace suggest that faith and spirituality can enhance inner peace since faith can guide individuals through difficult times.

**Table 1:** Pattern matrix: FACIT-Sp-12.

		Factors	
Items		Peace and meaning	Faith
1	I feel peaceful	0.739	
2	I have a reason for living	0.792	
3	My life has been productive	0.806	
4	I have trouble finding peace of mind		0.297
5	I feel a sense of purpose in my life	0.863	
6	I am able to reach deep into myself for comfort	0.829	
7	I feel a sense of harmony within myself	0.847	
8	My life lacks meaning and purpose		0.324
9	I find comfort in my faith or spiritual beliefs		0.813
10	I find strength in my faith or spiritual beliefs		0.828
11	My illness has strengthened my faith or spiritual beliefs		0.833
12	I know that whatever happens during COVID-19, things will be okay		0.408

A mean factor score of 14.86 (SD = 6.3) for the Meaning/Peace factor in the FACIT-SP-12 scale suggested that, on average, respondents experienced a lack of meaning and peace during the COVID-19 pandemic. This may be related to increased stress, uncertainty, and disruptions to daily life brought on by the pandemic, which may have posed a threat to individuals’ sense of purpose and inner peace.

The Faith factor’s mean score of 10.83 (SD = 5.21) suggests that, on average, respondents found solace and strength in their spiritual beliefs during this challenging period. This may have implied that spirituality served as a significant coping mechanism for many individuals during the pandemic, providing a source of comfort, resilience, and hope in the face of adversity. This suggested that respondents experienced purpose, tranquility, and faith at a level that is quite similar to the average.

### Associations between resilience and well-being

The associations between resilience (based on the CD-RISC 10) and well-being (general well-being based on the WHO-5, mental health based on the PMHS and spiritual well-being based on FACIT Sp-12) were further explored using Spearman’s rho. Spearman’s rho is an effective measure for assessing association, suitable for variables captured on an ordinal scale or higher and is applicable across any continuous bivariate distribution (Faherty, 2007; Salkind, 2007). To measure the strength of these associations, established guidelines were utilized. Specifically, a Spearman’s rho value exceeding 0.5 indicated a strong effect size, while a value of 0.3 signified a medium effect size, and a value of 0.1 indicated a small effect size (Salkind, 2007). These standardized criteria were adopted in this research to interpret the magnitude and significance of the relationships in this study.

A practically non-significant association (*r* = 0.241) was found between resilience and general well-being. The results revealed a positive association that leaned toward being practically significant between Resilience and Positive Mental Health (*r* = 0.382). This indicated that as the respondents tended to have higher levels of positive mental health during COVID-19 their resilience also tended to be higher. The association between Resilience and Meaning/Peace (FACIT1; *r* = 0.580) was practically significant and the association between Resilience and Faith (FACIT2; *r* = 0.361) leaned toward being practically significant. This association reflected that as respondents tended to have higher levels of spiritual well-being (both meaning/peace and faith) during COVID-19 they also tended to have higher levels of resilience (refer to Table 2 for correlations).

**Table 2:** Correlations between resilience and psychological well-being.

		Resilience	Well-being	Meaning and peace	Faith	Positive mental health
		CD-RISC 10	WHO-5	FACIT1	FACIT2	PMHS
Resilience	Correlation coefficient	1.000	0.241**	0.580**	0.361**	0.382**
CD-RISC 10	*p* value		0.000	0.000	0.000	0.000
Well-being	Correlation coefficient	0.241**	1.000	0.367**	0.205**	0.404**
WHO-5	*p* value	0.000		0.000	0.000	0.000
Meaning and peace	Correlation coefficient	0.580**	0.367**	1.000	0.543**	0.643**
FACIT1	*p* value	0.000	0.000		0.000	0.000
Faith	Correlation Coefficient	0.361**	0.205**	0.543**	1.000	0.426**
FACIT2	*p* value	0.000	0.000	0.000		0.000
Positive mental health	Correlation Coefficient	0.382**	0.404**	0.643**	0.426**	1.000
PMHS	*p* value	0.000	0.000	0.000	0.000	

**Correlation is significant at the 0.01 level (2-tailed).

## Discussion

There has been a growing interest in understanding and promoting human well-being globally, with researchers attempting to identify factors that promote and alter well-being (Tang et al., 2019; Ripp et al., 2020; Padmanabhanunni and Pretorius, 2023) This interest has been sparked by knowledge of mental health problems as well as a desire to comprehend and advance good mental health. This curiosity has also been driven by several factors, including a greater emphasis on sustainability and quality of life, a growing acknowledgement of the benefits of well-being, and an increased awareness of the importance of both mental and physical health. This study examined the relationship between resilience and psychological well-being by considering the well-being index of individuals as well as their positive mental health and spiritual well-being.

As previous studies have indicated, resilience is an important resource in the development of life satisfaction and promoting well-being (Di Fabio and Palazzeschi, 2015; Smith and Hollinger-Smith, 2015) and reduces anxiety, stress, and depression (Shi et al., 2015, 2022; Ortiz-Calvo et al., 2022). It is not surprising that resilience played an important role in the well-being of South African individuals during the COVID-19 pandemic. The pandemic has been a major source of stress and disruption worldwide, and individuals who were able to adapt and cope with the challenges of the pandemic were likely to experience better mental health and well-being. By focusing on positive emotions, maintaining a sense of purpose and meaning, and staying connected to their spirituality and faith, individuals were able to develop resilience and promote their own well-being. Individuals who have a sense of meaning and purpose in life and/or experience peace are more likely to have higher levels of resilience (Burrow and Patrick, 2020; Wilson et al., 2021). Findings from this study confirm that those with higher levels of meaning and purpose in their lives show greater resilience. Therefore, individuals who have a strong sense of meaning and purpose in life and/or experience peace may be better equipped to cope with stress and adversity, as they are more likely to have a positive outlook, a sense of direction, and a belief in their ability to overcome obstacles, which in turn develops resilience.

A person’s sense of belonging and their relationship to their creator (Kira and Tummala-Narra, 2017), as well as their sense of meaning, purpose, and values in life, all contribute to their subjective sense of spiritual well-being. It is a feeling of inner peace, contentment, and fulfillment that comes from living in line with one’s beliefs and ideals. Data from this study revealed that spirituality is positively associated with resilience. Hence, those with a high spirituality score had higher resilience. There is evidence to suggest that individuals who report higher levels of spirituality tend to experience greater levels of subjective well-being, lower levels of anxiety and depression, and better coping skills and resilience in the face of life stressors (Calamba and Magallanes, 2023; Hiles et al., 2023). Individuals with higher resilience were found to have a reduced risk of developing psychopathology (Koh Boon Yau et al., 2020).

According to the findings of a study which investigated resiliency and mental health, conducted on adults in Malaysia, it was discovered that resiliency significantly and favorably predicted positive mental health (Liew et al., 2021). Osimo et al. (2021) found that people living in Italy with higher levels of resilience had a positive response to the stressors connected to the pandemic (Osimo et al., 2021). Findings from a study conducted on college students also revealed that resilience can act as a buffer against psychological distress and contribute to improvements in well-being during pandemic periods (Luo et al., 2022). This suggests that resilience plays a protective function in determining individuals’ mental health during the pandemic.

Charles Darwin once said, “It is not the strongest of the species that survives, nor the most intelligent, but the one most responsive to change” (Darwin, 1859). Darwin, in his text, highlights the importance of adaptation and flexibility in the face of changing environments, which is a key concept in evolutionary biology. Darwin’s (1859) theory proposes that organisms that are better adapted to their environment have a higher chance of survival. The ability to adapt and respond to changing circumstances, rather than sheer strength or intelligence, is often what determines the success and survival of a species. During the COVID-19 pandemic, individuals had to adapt to a new lifestyle with added responsibilities such as childcare, maintaining a work-life balance, and managing their mental and physical health. Many people experienced significant changes in their daily routines and had to find ways to adapt to the new normal. This study proved that individuals were able to adapt their lifestyle to respond to the challenging circumstances of the pandemic and that their positive mental health and spirituality assisted with this adaptation, leading to resilient outcomes.

The results of this study confirmed that the COVID-19 pandemic had negative effects on the general population and that resilience may improve well-being. These findings concurred with most studies globally that indicate high levels of anxiety, stress, low mental health and depression due to the pandemic. Both resiliency and well-being are interrelated ideas that, when combined, can significantly enhance an individual’s overall mental and emotional health. The more we comprehend the connection between them, the easier it will be for us to devise methods to improve our health and deal with the challenges that life throws at us.

## Conclusion

Resilience and psychological well-being are strongly interconnected. Resilience is the ability to adapt and cope in the face of adversity, and it is a key factor that can promote positive mental health. More resilient individuals are better able to manage stress, maintain a positive outlook, and develop effective coping strategies. Individuals who have higher levels of resilience are more likely to experience positive emotions, maintain positive relationships, and have a sense of purpose and meaning in life. Additionally, resilient individuals are better equipped to manage difficult circumstances, such as the COVID-19 pandemic or other life stressors and maintain their overall well-being.

Cultivating resilience is an important component of promoting positive mental health. By developing effective coping strategies, maintaining social connections, and focusing on positive emotions, individuals can increase their resilience and better manage stress and adversity. Additionally, by promoting factors such as social support, positive relationships, coping skills, self-regulation, positive self-concept, meaning and purpose, and physical health, individuals can work toward achieving optimal well-being and positive mental health. By understanding the factors that promote resilience and developing strategies to cultivate it, individuals can better manage stress and adversity and maintain their overall well-being.

### Limitations

While research on mental health and resilience during COVID-19 has offered valuable understanding of how the pandemic has altered individuals’ mental health, several limitations to this research should be taken into consideration. These limitations include:

- This study’s findings may have limited generalizability to the broader South African population. The sample consisted only of individuals with access to a device and the Internet, excluding those in rural areas without such access. Consequently, the results may not fully represent the resilience and well-being of individuals in these underserved regions, thereby challenging the study’s external validity.
- Internal validity concerns arise from the possibility that some respondents had pre-existing mental health conditions. These conditions may have shaped their responses, complicating the task of distinguishing the pandemic’s role from pre-existing mental health issues. This factor restricts our capacity to make definitive causal conclusions regarding how COVID-19 has shaped mental health and resilience.
- Data collection in mid-2022 might not accurately capture the experiences and mental states of respondents during the pandemic’s peak in 2020–2021. This time lag may affect the internal validity of the study by introducing recall bias. Additionally, it raises questions about construct validity, as the constructs of mental health and resilience might have evolved or been shaped by factors other than the pandemic during this period.
- The study’s reliance on self-reported measures for mental health, resilience, and well-being introduces potential bias, changing its construct validity. Self-reports can be shaped by personal perceptions, memory recall, and social desirability, which may not accurately reflect the true mental health status of respondents.
- Given the cross-sectional nature of this study, it is important to note that causality cannot be established. The design limits our ability to determine whether the observed associations are causal in nature or the result of other confounding factors. This limitation significantly shapes the study’s internal validity in terms of establishing cause-and-effect relationships.
- This study utilized a non-probability convenience sampling method, selecting respondents through subjective, non-random means. This approach may limit the applicability of the findings to the wider population. Several potential sources of bias in this sampling method are noteworthy:

o Selection bias: The respondents in this convenience sample were not chosen through a random selection process. Instead, the sample consisted of individuals who were readily accessible and willing to engage in the study. This method may not accurately capture the diversity of the broader population, potentially skewing the results.
o Volunteer bias: The study depended on volunteer respondents. Those who opt to participate in research often have distinct characteristics or motivations from those who refrain from volunteering. Such differences can introduce bias in the findings.
o Demographic limitations: The study’s participation criteria required access to personal computers, smartphones, or tablets, along with internet connectivity. This prerequisite likely excluded contributions from individuals lacking such technology or internet access, especially those in remote or rural areas. Consequently, the sample may lack a full spectrum of demographic representation.
o Response bias influenced by compensation: Respondents were compensated for completing the questionnaire. While this practice aimed to acknowledge their time and effort, it might have influenced their motivation to participate. As a result, there’s a possibility that the responses may have been shaped by the incentive rather than reflecting genuine perspectives or experiences.

### Future recommendations

Resilience research during COVID-19 has provided valuable insights into the factors that promote resilience and well-being during times of stress and uncertainty. As we continue to navigate the ongoing challenges of the pandemic, there are several recommendations for future resilience research:

- Longitudinal studies: Future resilience research should incorporate longitudinal designs that track individuals’ resilience over time. This will allow researchers to better understand the long-term changes brought by the pandemic on resilience and mental health and to identify factors that promote resilience over time.
- Diverse samples: Resilience research should aim to include diverse samples that represent a range of demographic groups and populations. This will ensure that the findings apply to a wide range of individuals and communities.
- Multidisciplinary approaches: Resilience research should incorporate multidisciplinary approaches that draw on insights from fields such as psychology, sociology, public health, and neuroscience. This will allow researchers to develop a more comprehensive understanding of the factors that contribute to resilience and identify effective interventions for promoting it.

One cannot just recover from daily stressors, pandemics and losses. Rather, moving through them and moving forward is necessary. By transforming pain into strength and suffering into power, the dynamic process of resilience weaves the threads that will support recovery. Importantly, strength of character cannot be judged before an individual is tested by adversity. By developing a more comprehensive understanding of resilience and how it shapes mental health, researchers can identify effective strategies for promoting well-being and resilience during times of stress and uncertainty.

## Data availability statement

The datasets presented in this article are not readily available because this study formed part of a larger PhD study. Requests to access the datasets should be directed to 20062621@nwu.ac.za.

## Ethics statement

The studies involving humans were approved by Human Research Ethics Committee of the North-West University. The studies were conducted in accordance with the local legislation and institutional requirements. The participants provided their written informed consent to participate in this study.

## Author contributions

TS: Writing – original draft, Writing – review & editing. HM: Supervision, Writing – review & editing. EF: Formal analysis, Writing – review & editing.

## References

[ref1] Aktürk Ü. Erci B. Araz M. (2017). Functional evaluation of treatment of chronic disease: validity and reliability of the Turkish version of the spiritual well-being scale. Palliat. Support. Care 15, 684–692. doi: 10.1017/S1478951517000013, PMID: 28183363

[ref2] Almubaddel A. (2022). Psychometric properties of a Saudi Arabian version of the positive mental health (PMH) scale. Psicol-Reflecx. Crit. 35:29. doi: 10.1186/s41155-022-00232-0, PMID: 36125579 PMC9489822

[ref3] Aluh D. O. Onu J. U. (2020). The need for psychosocial support amid COVID-19 crises in Nigeria. Psychol. Trauma Theory Res. Pract. Policy 12, 557–558. doi: 10.1037/tra0000704, PMID: 32567871

[ref4] Aruta J. J. B. R. (2022). Socio-ecological determinants of distress in Filipino adults during COVID-19 crisis. Curr. Psychol. 41, 7482–7492. doi: 10.1007/s12144-020-01322-x, PMID: 33424204 PMC7783297

[ref5] Baluku M. M. Bantu E. Namale B. Otto K. (2022). Maintaining high Eudaimonic wellbeing despite ambiguity intolerance among three employment status groups: examining the buffering effects of positive psychological attributes. Int. J. Appl. Posit. Psychol. 7, 1–30. doi: 10.1007/s41042-021-00051-1, PMID: 33816777 PMC8008017

[ref6] Baños R. M. Garcés J. J. Miragall M. Herrero R. Vara M. D. Soria-Olivas E. (2021). Exploring the heterogeneity and trajectories of positive functioning variables, emotional distress, and post-traumatic growth during strict confinement due to COVID-19. J. Happiness Stud. 23, 1683–1708. doi: 10.1007/s10902-021-00469-z, PMID: 34744499 PMC8561082

[ref7] Beames J. R. Li S. H. Newby J. M. Maston K. Christensen H. Werner-Seidler A. (2021). The upside: coping and psychological resilience in Australian adolescents during the COVID-19 pandemic. Child Adolesc. Psychiatry Ment. Health 15, 77–10. doi: 10.1186/s13034-021-00432-z, PMID: 34922575 PMC8684334

[ref8] Boitshwarelo T. Koen M. Rakhudu M. (2022). Strategies to enhance the resilience of nurse managers. Africa J. Nurs. Midwifery. 24, 1–21. doi: 10.25159/2520-5293/8888

[ref9] Boufellous S. Sánchez-Teruel D. Robles-Bello M. A. Lorabi S. Mendoza-Bernal I. Lara-Cabrera M. L.. (2023). Psychometric properties of the positive mental health scale in a Spanish population. SAGE Open 13:215824402311727. doi: 10.1177/21582440231172743

[ref10] Brady M. J. Peterman A. H. Fitchett G. Mo M. Cella D. (1999). A case for including spirituality in quality of life measurement in oncology. Psychooncology 8, 417–428. doi: 10.1002/(SICI)1099-1611(199909/10)8:5<417::AID-PON398>3.0.CO;2-4 10559801

[ref11] Brailovskaia J. Margraf J. (2020). Predicting adaptive and maladaptive responses to the coronavirus (COVID-19) outbreak: a prospective longitudinal study. Int. J. Clin. Health Psychol. 20, 183–191. doi: 10.1016/j.ijchp.2020.06.002, PMID: 32837518 PMC7321043

[ref1006] Bredle J. M. Salsman J. M. Debb S. M. Arnold B. J. Cella D. (2011). Spiritual well-being as a component of health-related quality of life: The Functional Assessment of Chronic Illness Therapy-Spiritual Well-Being Scale (FACIT-Sp). Religions 2, 77–94.

[ref12] Burrow A. L. Patrick L. H. (2020). The Ecology of Purposeful Living Across the Lifespan. Cham: Springer

[ref13] Calamba P. C. Magallanes C. I. (2023). Spiritual well-being and resilience of emerging adults of a Catholic College in Central Negros. Tech. Soc. Sci. J. 41, 169–183. doi: 10.47577/tssj.v41i1.8592

[ref14] Campbell-Sills L. Stein M. B. (2007). Psychometric analysis and refinement of the connor–Davidson resilience scale (CD‐RISC): validation of a 10‐item measure of resilience. J. Trauma. Stress. 20, 1019–1028. doi: 10.1002/jts.20271, PMID: 18157881

[ref15] Carranza Esteban R. F. Mamani-Benito O. Cjuno J. Tito-Betancur M. Lingán-Huamán S. K. Arias-Chávez D. (2022). Translation and validation of the WHO-5 general well-being index into native language Quechua of the Peruvian south. SSRN Electron. J. 9:e16843. doi: 10.2139/ssrn.4204317 PMC10258449 37313139

[ref16] Casali N. Feraco T. Meneghetti C. (2021). Character strengths sustain mental health and post-traumatic growth during the COVID-19 pandemic: a longitudinal analysis. Psychol. Health 37, 1663–1679. doi: 10.1080/08870446.2021.1952587, PMID: 34288790

[ref17] Cavazos Vela J. Castro V. Cavazos L. Cavazos M. Gonzalez S. L. (2014). Understanding Latina/o students’ meaning in life, spirituality, and subjective happiness. J. Hisp. High. Educ. 14, 171–184. doi: 10.1177/1538192714544524

[ref18] Chan L. Liu R. K. W. Lam T. P. Chen J. Y. Tipoe G. L. Chan L., et.al. (2022). Validation of the World Health Organization well-being index (WHO-5) among medical educators in Hong Kong: a confirmatory factor analysis. Med. Educ. 27:2044635. doi: 10.1080/10872981.2022.2044635, PMID: 35275804 PMC8920356

[ref19] Cobo-Cuenca A. I. Fernández-Fernández B. Carmona-Torres J. M. Pozuelo-Carrascosa D. P. Laredo-Aguilera J. A. Romero-Gómez B.. (2022). Longitudinal study of the mental health, resilience, and post-traumatic stress of senior nursing students to nursing graduates during the COVID-19 pandemic. Int. J. Environ. Res. Public Health 19:13100. doi: 10.3390/ijerph192013100, PMID: 36293681 PMC9602859

[ref20] Connor K. Davidson J. R. T. (2003). Development of a new resilience scale: the Connor-Davidson resilience scale (CD-RISC). Depress. Anxiety 18, 76–82. doi: 10.1002/da.10113 12964174

[ref21] Cosma A. Költő A. Chzhen Y. Kleszczewska D. Kalman M. Martin G. (2022). Measurement invariance of the WHO-5 well-being index: evidence from 15 European countries. Int. J. Environ. Res. Public Health 19:9798. doi: 10.3390/ijerph19169798, PMID: 36011429 PMC9407912

[ref22] Damen A. Visser A. Van Laarhoven H. W. M. Leget C. Raijmakers N. Van Roij J.. (2021). Validation of the FACIT-Sp-12 in a Dutch cohort of patients with advanced cancer. Psycho-Oncology 30, 1930–1938. doi: 10.1002/pon.5765, PMID: 34258819

[ref23] Darwin C. (1859). On the Origin of Species by Means of Natural Selection, or the Preservation of Favoured Races in the Struggle for Life. London: John Murray. PMC5184128 30164232

[ref24] Davidson J. R. T. (2020). Connor-Davidson resilience scale (CD-RISC): © manual. Available at: http://www.cd-risc.com/ (Accessed February 2, 2023).

[ref25] Di Fabio A. Palazzeschi L. (2015). Hedonic and eudaimonic well-being: the role of resilience beyond fluid intelligence and personality traits. Front. Psychol. 6:1367. doi: 10.3389/fpsyg.2015.01367, PMID: 26441743 PMC4566034

[ref26] Di Giuseppe M. Nepa G. Prout T. A. Albertini F. Marcelli S. Orrù G.. (2021). Stress, burnout, and resilience among healthcare workers during the covid-19 emergency: the role of defense mechanisms. Int. J. Environ. Res. Public Health 18:5258. doi: 10.3390/ijerph18105258, PMID: 34069270 PMC8156145

[ref27] Didkowsky N. K. Ungar M. (2016). “A social eceological approach to understanding resilience among rural youth” in The Routledge International Handbook of Psychosocial Resilience. ed. Kumar U. (London: Routledge)

[ref28] Dodge R. Daly A. P. Huyton J. Sanders L. D. (2012). The challenge of defining wellbeing. Int. J. Wellbeing 2, 222–235. doi: 10.25273/counsellia.v9i1.3209

[ref29] Dubus N. (2018). Integration or building resilience: what should the goal be in refugee resettlement? J. Immigr. Refug. Stud. 16, 413–429. doi: 10.1080/15562948.2017.1358409

[ref30] Fabrigar L. R. Wegener D. T. (2011). Exploratory Factor Analysis. Oxford, UK: Oxford University Press

[ref31] Faherty V. (2007). Compassionate Statistics: Applied Quantitative Analysis for Social Services (With Exercises and Instructions in SPSS). Los Angeles, CA: Sage

[ref32] Field A. (2005). Discovering Statistics Using SPSS: (and Sex and Drugs and Rock “n” Roll). (2nd Edn.). London: Sage

[ref33] Field A. (2009). Discovering Statistics Using SPSS. London: Sage

[ref34] Field A. (2013). Discovering Statistics Using IBM SPSS Statistics. London: Sage

[ref35] Finch W. H. (2020). Using fit statistic differences to determine the optimal number of factors to retain in an exploratory factor analysis. Educ. Psychol. Meas. 80, 217–241. doi: 10.1177/0013164419865769, PMID: 32158020 PMC7047263

[ref36] Finlay J. M. Kler J. S. O’Shea B. Q. Eastman M. R. Vinson Y. R. Kobayashi L. C. (2021). Coping during the COVID-19 pandemic: a qualitative study of older adults across the United States. Front. Public Health 9:643807. doi: 10.3389/fpubh.2021.643807, PMID: 33898379 PMC8058195

[ref37] Fredrickson B. L. (1998). What good are positive emotions? Rev. Gen. Psychol. 2, 300–319. doi: 10.1037/1089-2680.2.3.300, PMID: 21850154 PMC3156001

[ref38] Friborg O. Hjemdal O. Rosenvinge J. H. Martinussen M. (2003). A new rating scale for adult resilience: what are the central protective resources behind healthy adjustment? Int. J. Methods Psychiatr. Res. 12, 65–76. doi: 10.1002/mpr.143, PMID: 12830300 PMC6878238

[ref39] Goldberg A. E. Mccormick N. Virginia H. (2021). Parenting in a pandemic: work—family arrangements, well-being, and intimate relationships among adoptive parents. Fam. Relat. 70, 7–25. doi: 10.1111/fare.12528

[ref40] Gonzalez-Mendez R. Ramírez-Santana G. Hamby S. (2021). Analyzing Spanish adolescents through the Lens of the resilience portfolio model. J. Interpers. Violence 36, 4472–4489. doi: 10.1177/088626051, PMID: 30071767

[ref41] Hair J. F. Babin B. J. Anderson R. E. Black W. C. (2019). Multivariate Data Analysis. 8th Edn Cengage. England: Pearson Prentice.

[ref42] Hiles A. Megan H. Tony R. Nicole M. Wilke G. (2023). The relationship between spirituality and resilience and well—being: a study of 529 care leavers from 11 nations. Advers. Resil. Sci. 4, 177–190. doi: 10.1007/s42844-023-00088-y, PMID: 36816809 PMC9918825

[ref43] Hurtes K. P. Allen L. R. (2001). Measuring resiliency in youth: the resiliency attitudes skills profile. Ther. Recreat. J. 35, 333–347.

[ref44] Huta V. Ryan R. M. (2010). Pursuing pleasure or virtue: the differential and overlapping well-being benefits of he-donic and eudemonic motives. J. Happiness Stud. 11, 735–762. doi: 10.1007/s10902-009-9171-4

[ref45] Huta V. Waterman A. S. (2014). Eudaimonia and its distinction from Hedonia: developing a classification and terminology for understanding conceptual and operational definitions. J. Happiness Stud. 15, 1425–1456. doi: 10.1007/s10902-013-9485-0

[ref46] Kassab Alshayea A. (2023). Development and validation of an Arabic version of the World Health Organization well-being index (WHO-5). J. Psychopathol. Behav. Assess. 45, 247–255. doi: 10.1007/s10862-023-10027-x

[ref47] Kavčič T. Avsec A. Kocjan G. Z. (2020). Psychological functioning of Slovene adults during the COVID-19 pandemic: does resilience matter? Psychiatry Q. 92, 207–216. doi: 10.1007/s11126-020-09789-4, PMID: 32556914 PMC7299145

[ref48] Kavčič T. Zager Kocjan G. Dolenc P. (2021). Measurement invariance of the CD-RISC-10 across gender, age, and education: a study with Slovenian adults. Curr. Psychol. 42, 1727–1737. doi: 10.1007/s12144-021-01564-3, PMID: 33723479 PMC7945969

[ref49] Kira I. A. Tummala-Narra P. (2017). Psychotherapy with refugees: emerging paradigm psychotherapy with refugees: emerging paradigm. J. Loss Trauma 20, 449–467. doi: 10.1080/15325024.2014.949145

[ref50] Koh Boon Yau E. Pang Tze Ping N. Shoesmith W. D. James S. Nor Hadi N. M. Loo J. L. (2020). The behaviour changes in response to COVID-19 pandemic within Malaysia. Malaysian J. Med. Sci. 27, 45–50. doi: 10.21315/mjms2020.27.2.5, PMID: 32788840 PMC7409570

[ref51] Lara-Cabrera M. L. Betancort M. Muñoz-Rubilar A. Rodríguez-Novo N. Bjerkeset O. De Las Cuevas C. (2022). Psychometric properties of the WHO-5 well-being index among nurses during the COVID-19 pandemic: a cross-sectional study in three countries. Int. J. Environ. Res. Public Health 19:10106. doi: 10.3390/ijerph191610106, PMID: 36011741 PMC9407690

[ref52] Li L. Mao M. Wang S. Yin R. Yan H. Jin Y.. (2022). Posttraumatic growth in Chinese nurses and general public during the COVID-19 outbreak. Psychol. Health Med. 27, 301–311. doi: 10.1080/13548506.2021.1897148, PMID: 33726576

[ref53] Liew E. W. K. Low E. M. J. Ho G. L. L. T'ng S. T. Ho K. H. (2021). Perceived risk, fear of Covid-19, and resilience on mental health of Malaysian emerging adults during the Covid-19 pandemic. Int. J. Res. Counsel. Educ. 5, 152–164. doi: 10.24036/00456za0002

[ref54] Low K. Y. Pheh K. S. Tan C. S. (2023). Validation of the WHO-5 as a screening tool for depression among young adults in Malaysia. Curr. Psychol. 42, 7841–7844. doi: 10.1007/s12144-021-02152-1

[ref55] Lukat J. Margraf J. Lutz R. Der Veld W. M. Becker E. S. (2016). Psychometric properties of the positive mental health scale (PMH-scale). BMC Psychol. 4, 1–14. doi: 10.1186/s40359-016-0111-x, PMID: 26865173 PMC4748628

[ref56] Luo C. Santos-Malave G. Taku K. Katz C. Yanagisawa R. (2022). Post-traumatic growth and resilience among American medical students during the COVID-19 pandemic. Psychiatry Q. 93, 599–612. doi: 10.1007/s11126-022-09981-8, PMID: 35211827 PMC8870080

[ref57] Luthar S. S. (ed.). (2003). Resilience and Vulnerability: Adaptation in the Context of Childhood Adversities. New York: Cambridge University Press

[ref58] Luthar S. S. (2006). “Resilience in development: a synthesis of research across five decades” in Developmental Psychopathology: Risk, Disorder and Adaptation. eds. Cicchetti D. Cohen D. J. (Hoboken, NJ: Wiley), 739–795.

[ref59] Luthar S. S. Chicchetti D. Becker B. (2000). The construct of resilience: a critical evaluation and guidelines for future work. Child Dev. 71, 543–562. doi: 10.1111/1467-8624.00164, PMID: 10953923 PMC1885202

[ref60] Lyubomirsky S. L. King L. Diener E. (2005). The benefits of frequent positive affect: does happiness lead to success? Psychol. Bull. 131, 803–855. doi: 10.1037/0033-2909.131.6.803, PMID: 16351326

[ref61] Maragha T. Donnelly L. Schuetz C. Von Bergmann H. C. Brondani M. (2023). Students’ resilience and mental health in the dental curriculum. Eur. J. Dent. Educ. 27, 174–180. doi: 10.1111/eje.12790, PMID: 35178840

[ref62] Masten A. S. (2007). Resilience in developing systems: Progress and promise as the fourth wave rises. Dev. Psychopathol. 19, 921–930. doi: 10.1017/S0954579407000442, PMID: 17705908

[ref63] Meyers L. S. Gamst G. C. Guarino A. J. (2013). Performing Data Analysis Using IBM SPSS. New Jersey: John Wiley & Sons.

[ref64] Miragall M. Herrero R. Vara M. D. Galiana L. Baños R. M. (2021). The impact of strict and forced confinement due to the COVID-19 pandemic on positive functioning variables, emotional distress, and posttraumatic growth in a Spanish sample. Eur. J. Psychotraumatol. 12:1918900. doi: 10.1080/20008198.2021.1918900, PMID: 34178293 PMC8205045

[ref65] Monod S. Lécureux E. Rochat E. Spencer B. Seematter-bagnoud L. Büla C. (2015). Validity of the FACIT-Sp to assess spiritual well-being in elderly Patient. Eur. J. Dent. Educ. 6, 1311–1322. doi: 10.4236/psych.2015.610128

[ref66] Mosheva M. Hertz-Palmor N. Dorman Ilan S. Matalon N. Pessach I. M. Afek A.. (2020). Anxiety, pandemic-related stress and resilience among physicians during the COVID-19 pandemic. Depress. Anxiety 37, 965–971. doi: 10.1002/da.23085, PMID: 32789945 PMC7436709

[ref67] Ngoc Nguyen B. T. Huynh S. Van Nguyen T. N. Nguyen-Duong B. T. Ngo-Thi T. T. Tran-Chi V. L. (2022). Mediation effects of post-series depression on the relationship between life satisfaction and positive mental health of Vietnamese: a cross-sectional study in COVID-19 pandemic context. Front. Psychol. 13:971711. doi: 10.3389/fpsyg.2022.971711, PMID: 36518965 PMC9744194

[ref1005] October K. R. Petersen L. R. Adebiyi B. Rich E. Roman N. V. (2022). COVID-19 daily realities for families: A South African sample. International Journal of Environmental Research and Public Health 19:221. doi: 10.3390/ijerph19010221 PMC8750582 35010480

[ref68] Ortiz-Calvo E. Martínez-Alés G. Mediavilla R. González-Gómez E. Fernández-Jiménez E. Bravo-Ortiz M. F.. (2022). The role of social support and resilience in the mental health impact of the COVID-19 pandemic among healthcare workers in Spain. J. Psychiatr. Res. 148, 181–187. doi: 10.1016/j.jpsychires.2021.12.030, PMID: 35124398 PMC8668396

[ref69] Osimo S. A. Aiello M. Gentili C. Ionta S. Cecchetto C. (2021). The influence of personality, resilience, and alexithymia on mental health during COVID-19 pandemic. Front. Psychol. 12:630751. doi: 10.3389/fpsyg.2021.630751, PMID: 33716896 PMC7943855

[ref70] Padmanabhanunni A. Pretorius T. B. (2023). Promoting well-being in the face of a pandemic: the role of sense of coherence and ego-resilience in the relationship between psychological distress and life satisfaction. S. Afr. J. Psychol. 53, 124–133. doi: 10.1177/00812463221113671, PMID: 37038457 PMC10076959

[ref71] Pallant J. (2013). SPSS Survival Manual: A Step-by-Step Guide to Data Analysis Using IBM SPSS (5th Edn.) New York, NY: McGraw-Hill

[ref72] Park C. L. Finkelstein-Fox L. Russell B. S. Fendrich M. Hutchison M. Becker J. (2021). Psychological resilience early in the COVID-19 pandemic: stressors, resources, and coping strategies in a National Sample of Americans. Am. Psychol. 76, 715–728. doi: 10.1037/amp0000813, PMID: 34081505 PMC8595499

[ref73] Peterman A. Fitchett G. Brady L. M.. (2002). Meapoallsuring spiritual well-being in people with cancer: the functional assessment of chronic illness therapy–spiritual well-being scale. Ann. Behav. Med. 24, 49–58. doi: 10.1207/S15324796ABM2401_06 12008794

[ref74] Peterson C. Seligman M. E. P. (2003). Character strengths before and after September 11. Psychol. Sci. 14, 381–384. doi: 10.1111/1467-9280.24482, PMID: 12807415

[ref75] Petry S. E. Hughes D. Galanos A. (2020). Grief: the epidemic within an epidemic. Am. J. Hospice Palliat. Care 38, 419–422. doi: 10.1177/1049909120978796, PMID: 33280398 PMC7723733

[ref76] Pillay D. (2020). Positive affect and mindfulness as predictors of resilience amongst women leaders in higher education institutions. SA J. Hum. Resour. Manag. 18:a1260. doi: 10.4102/sajhrm.v18i0.1260

[ref77] Pleeging E. Burger M. Van Exel J. (2021). The relations between Hope and subjective well-being: a literature overview and empirical analysis. Appl. Res. Qual. Life 16, 1019–1041. doi: 10.1007/s11482-019-09802-4

[ref78] Polizzi C. Lynn S. J. Perry A. (2020). Stress and coping in the time of Covid-19: pathways to resilience and recovery. Clin. Neuropsychiatry 17, 59–62. doi: 10.36131/CN20200204, PMID: 34908968 PMC8629051

[ref79] Quoidbach J. Berry E. V. Hansenne M. Mikolajczak M. (2010). Positive emotion regulation and well-being: comparing the impact of eight savoring and dampening strategies. Personal. Individ. Differ. 49, 368–373. doi: 10.1016/j.paid.2010.03.048

[ref80] Rabitti E. Cavuto S. Iani L. Ottonelli S. De Vincenzo F. Costantini M. (2020). The assessment of spiritual well-being in cancer patients with advanced disease: which are its meaningful dimensions? BMC Palliat. Care 19, 1–8. doi: 10.1186/s12904-020-0534-2, PMID: 32143609 PMC7059276

[ref81] Rahmani K. Gnoth J. Mather D. (2018). Hedonic and eudaimonic well-being: a psycholinguistic view. Tour. Manag. 69, 155–166. doi: 10.1016/j.tourman.2018.06.008

[ref82] Rajan S. K. Harifa P. R. Pienyu R. (2017). Academic resilience, locus of control, academic engagement and self-efficacy among the school children. Ind. J. Posit. Psychol. 8, 507–511.

[ref83] Raniga T. Mthembu M. (2017). Family resilience in low income communities: a case study of an informal settlement in KwaZulu-Natal, South Africa. Int. J. Soc. Welf. 26, 276–284. doi: 10.1111/ijsw.12243

[ref84] Rich E. G. Butler-Kruger L. Sonn I. K. Kader Z. Roman N. V. (2022). Family resilience and the COVID-19 pandemic: a south African study. Soc. Sci. 11:416. doi: 10.3390/socsci11090416

[ref85] Ripp J. Peccoralo L. Charney D. (2020). Attending to the emotional well-being of the health care workforce in a new York City health system during the COVID-19 pandemic. Acad. Med. 95, 1136–1139. doi: 10.1097/ACM.0000000000003414, PMID: 32282344 PMC7176260

[ref86] Robinson M. D. Eid M. (2017). “Introduction to the happy mind: cognitive contributions to well-being” in The Happy Mind: Cognitive Contributions to Well-Being. eds. Robinson M. D. Eid M. (Switzerland: Springer International Publishing/Springer Nature), 1–19.

[ref87] Robles-Bello M. A. Sánchez-Teruel D. Valencia Naranjo N. (2020). Variables protecting mental health in the Spanish population affected by the COVID-19 pandemic. Curr. Psychol. 41, 5640–5651. doi: 10.1007/s12144-020-01132-1, PMID: 33106742 PMC7578437

[ref88] Rojas Flóres L. F. (2015). Factors affecting academic resilience in middle school students: a case study 1. GIST Educ. Learn. Res. J. 11, 63–78. doi: 10.26817/16925777.286

[ref89] Rolland J. S. (2020). COVID-19 pandemic: applying a multisystemic Lens. Fam. Process 59, 922–936. doi: 10.1111/famp.12584, PMID: 32677711 PMC7404743

[ref90] Ruggeri K. Garcia-Garzon E. Maguire Á. Matz S. Huppert F. A. (2020). Well-being is more than happiness and life satisfaction: a multidimensional analysis of 21 countries. Health Qual. Life Outcomes 18, 1–16. doi: 10.1186/s12955-020-01423-y, PMID: 32560725 PMC7304199

[ref91] Rutter M. (1993). “Resilience: some conceptual considerations” in Social Work: A Reader. eds. Cree V. E. McCulloch T. (Hoboken, NY: Taylor & Francis), 122–127.

[ref92] Ryan R. M. Deci E. L. (2001). On happiness and human potentials: a review of research on hedonic and eudaimonic well-being. Annu. Rev. Psychol. 52, 141–166. doi: 10.1146/annurev.psych.52.1.141, PMID: 11148302

[ref93] Ryff C. D. (1989). Happiness is everything, or is it? Explorations on the meaning of psychological well-being. J. Pers. Soc. Psychol. 57, 1069–1081. doi: 10.1037//0022-3514.57.6.1069

[ref94] Salkind N. J. (ed.) (2007). Encyclopedia of Measurement and Science 1-0. Thousand Oaks, Calif.: Sage

[ref1004] Sandberg S. Grant A. (2017). Option B: Facing adversity, building resilience, and finding joy. New York: Knopf.

[ref95] Seligman M. (2011). Flourish. New York, NY: Free Press

[ref96] Shi M. Wang X. Bian Y. Wang L.. (2015). The mediating role of resilience in the relationship between stress and life satisfaction among Chinese medical students: a cross-sectional study. BMC Med. Educ. 15, 1–7. doi: 10.1186/s12909-015-0297-2, PMID: 25890167 PMC4332721

[ref97] Shi L. S. B. Xu R. H. Xia Y. Chen D. X. Wang D. (2022). The impact of COVID-19-related work stress on the mental health of primary healthcare workers: the mediating effects of social support and resilience. Front. Psychol. 12:800183. doi: 10.3389/fpsyg.2021.800183, PMID: 35126252 PMC8814425

[ref98] Shrestha N. (2021). Factor analysis as a tool for survey analysis. Am. J. Appl. Math. Stat. 9, 4–11. doi: 10.12691/ajams-9-1-2

[ref99] Smith B. W. Dalen J. Wiggins K. Tooley E. Christopher P. Bernard J. (2008). The brief resilience scale: assessing the ability to bounce back. Int. J. Behav. Med. 15, 194–200. doi: 10.1080/10705500802222972, PMID: 18696313

[ref100] Smith J. L. Hollinger-Smith L. (2015). Savoring, resilience, and psychological well-being in older adults. Aging Ment. Health 19, 192–200. doi: 10.1080/13607863.2014.986647, PMID: 25471325

[ref1001] Snyder C. R. Lopez S. J. (eds.). (2002). Handbook of positive psychology. Oxford: Oxford University Press.

[ref101] Staehr J.K. (1998). The use of well-being measures in primary health care—the DepCare project. World Health Organisation regional Office for Europe: Well-being measures in primary health care-the DepCare project. Geneva: World Health Organisation.

[ref102] Steger M. F. Kashdan T. B. Oishi S. (2008). Being good by doing good: daily eudaimonic activity and well-being. J. Res. Pers. 42, 22–42. doi: 10.1016/j.jrp.2007.03.004

[ref103] Sümen A. Adibelli D. (2021). The effect of coronavirus (COVID-19) outbreak on the mental well-being and mental health of individuals. Perspect. Psychiatr. Care 57, 1041–1051. doi: 10.1111/ppc.12655 33103787

[ref104] Tang Y. Y. Tang R. Gross J. J. (2019). Promoting psychological well-being through an evidence-based mindfulness training program. Front. Hum. Neurosci. 13:237. doi: 10.3389/fnhum.2019.00237, PMID: 31354454 PMC6635568

[ref105] Tavakol M. Wetzel A. (2020). Factor analysis: a means for theory and instrument development in support of construct validity. Int. J. Med. Educ. 11, 245–247. doi: 10.5116/ijme.5f96.0f4a, PMID: 33170146 PMC7883798

[ref106] Toledano-Toledano F. Jiménez S. Moral de la Rubia J. Merino-Soto C. Rivera-Rivera L. (2023). Positive mental health scale (PMHS) in parents of children with Cancer: a psychometric evaluation using item response theory. Cancer 15:2744. doi: 10.3390/cancers15102744, PMID: 37345081 PMC10216644

[ref107] Ungar M. (2011). The social ecology of resilience: addressing contextual and cultural ambiguity of a nascent construct. Am. J. Orthop. 81, 1–17. doi: 10.1111/j.1939-0025.2010.01067.x, PMID: 21219271

[ref108] Ungar M. (2018). Systemic resilience: principles and processes for a science of change in contexts of adversity. Ecol. Soc. 23:34. doi: 10.5751/ES-10385-230434

[ref109] Velten J. Brailovskaia J. Margraf J. (2021). Positive mental health scale: validation and measurement invariance across eight countries, genders, and age groups. Psychol. Assess. 34, 332–340. doi: 10.1037/pas0001094, PMID: 34843278

[ref110] Wagnild G. M. Young H. M. (1993). Development and psychometric evaluation of the resilience scale. J. Nurs. Meas. 1, 165–178. PMID: 7850498

[ref111] Walsh F. (2020). Loss and resilience in the time of COVID-19: meaning making, Hope, and transcendence. Fam. Process 59, 898–911. doi: 10.1111/famp.12588, PMID: 32678915 PMC7405214

[ref112] Watts C. J. Hilliard R. C. Graupensperger S. (2022). Relationships between resilience, mental well-being, and COVID-19 worries in collegiate student-athletes. Front. Sports Active Living 4:890006. doi: 10.3389/fspor.2022.890006, PMID: 35647541 PMC9130569

[ref113] Wilson S. R. Kuang K. Hintz E. A. Buzzanell P. M. (2021). Developing and validating the communication resilience processes scale. J. Commun. 71, 478–513. doi: 10.1093/joc/jqab013

[ref114] Windle G. Markland D. A. Woods B. (2008). Examination of a theoretical model of psychological resilience in older age. Aging Ment. Health 12, 285–292. doi: 10.1080/13607860802120763, PMID: 18728940

[ref1002] Worldometer. (2023). COVID-19 Coronavirus Outbreak. https://www.worldometers.info/coronavirus/

[ref1003] World Health Organization (WHO). (2021). COVID-19 Response in South Africa: Country brief. WHO. Retrieved June, 5 2022, from https://www.afro.who.int/sites/default/files/2021-10/WHO%20Covid-19%20Response%20in%20South%20Africa_Country%20Brief.pd

[ref115] Yeung N. C. Y. Huang B. Lau C. Y. K. Lau J. T. F. (2022). Finding the silver linings in the COVID-19 pandemic: psychosocial correlates of adversarial growth among Filipina domestic helpers in Hong Kong. Psychol. Trauma Theory Res. Pract. Policy 14, 291–300. doi: 10.1037/tra0001069, PMID: 34435818

[ref116] Yıldırım M. Arslan G. Wong P. T. P. (2022). Meaningful living, resilience, affective balance, and psychological health problems among Turkish young adults during coronavirus pandemic. Curr. Psychol. 41, 7812–7823. doi: 10.1007/s12144-020-01244-8, PMID: 33424205 PMC7785475

